# Selenium levels and glutathione peroxidase activity in patients with ataxia-telangiectasia: association with oxidative stress and lipid status biomarkers

**DOI:** 10.1186/s13023-021-01732-5

**Published:** 2021-02-12

**Authors:** Itana Gomes Alves Andrade, Fabíola Isabel Suano-Souza, Fernando Luiz Affonso Fonseca, Carolina Sanchez Aranda Lago, Roseli Oselka Saccardo Sarni

**Affiliations:** 1 Department of Pediatrics, Universidade Federal de São Paulo, Escola Paulista de Medicina, Rua Dr. Diogo de Faria, 671, São Paulo, SP CEP 04037002 Brazil; 2 Universidade Federal de São Paulo, Campus Diadema, São Paulo, SP Brazil; 3 Department of Pediatrics, Faculdade de Medicina ABC/Centro Universitário Saúde ABC, Santo André, SP Brazil

**Keywords:** Ataxia-telangiectasia, Primary immunodeficiency, Dyslipidemia, Oxidative stress, Cardiovascular risk, Selenium

## Abstract

**Introduction:**

Ataxia-Telangiectasia (A-T) is a multi-system disorder that may be associated with endocrine changes, oxidative stress in addition to inflammation. Studies suggest that selenium is a trace element related to protection against damage caused by oxidative stress.

**Objective:**

To describe the plasma levels of selenium and erythrocyte glutathione peroxidase activity in A-T patients and to relate them to oxidative stress and lipid status biomarkers.

**Methods:**

This is a cross-sectional and controlled study evaluating 22 A-T patients (age median, 12.2 years old) matched by gender and age with 18 healthy controls. We evaluated: nutritional status, food intake, plasma selenium levels, erythrocyte glutathione peroxidase activity, lipid status, inflammation and oxidative stress biomarkers.

**Results:**

Adequate levels of selenium were observed in 24/36 (66.7%) in this evaluated population. There was no statistically significant difference between the groups in selenium levels [47.6 μg/L (43.2–57.0) vs 54.6 (45.2–62.6) μg/dL, *p* = 0.242]. Nine of A-T patients (41%) had selenium levels below the reference value. The A-T group presented higher levels of LDL-c, non-HDL-c, oxidized LDL, Apo B, Apo-B/Apo-A-I1, LDL-c/HDL-c ratio, malondialdehyde [3.8 µg/L vs 2.8 µg/L, *p* = 0.029] and lower Apo-A-I1/HDL-c and glutathione peroxidase activity [7300 U/L vs 8686 U/L, *p* = 0.005]. Selenium levels were influenced, in both groups, independently, by the concentrations of oxidized LDL, malonaldehyde and non-HDL-c. The oxidized LDL (AUC = 0.849) and ALT (AUC = 0.854) were the variables that showed the greatest discriminatory power between groups.

**Conclusion:**

In conclusion, we observed the presence of selenium below the reference value in nearly 40% and low GPx activity in A-T patients. There was a significant, inverse and independent association between selenium concentrations and oxidative stress biomarkers. Those data reinforce the importance of assessing the nutritional status of selenium in those patients.

## Introduction

Selenium (Se) is an essential micronutrient for antioxidant defense that integrates an important part of selenoproteins [1]. The most well-known selenoprotein is Glutathione peroxidase (GPx), which protects cells from damage caused by free radicals such as reactive oxygen species (ROS) [2, 3]. Seleniumblocks the activation of the nuclear transcription factor NFkB, a sensitive regulator that modulates the production of inflammatory mediators and adhesion molecules [4].

The trace element also stands out for its participation in immunological competence, particularly in cell-mediated immunity, cognitive function, and protection against cardiovascular diseases (CVDs) [5, 6].

Selenium, essentially needed for the biosynthesis of selenoproteins, e.g.GPx, that are involved in antioxidative defense systems and lipid metabolism. However, the associations between serum Se concentrations and the risk of dyslipidemia are still controversial in the literature [2].

Some scientific evidence has shown that Se deficiency occasionally increases the concentrations of total cholesterol (TC) [6] and triglycerides (TG) [7], elevating the risk of heart disease (HD), such as atherosclerosis, up to three times [8].

Ataxia-telangiectasia (A-T) or Louis–Bar syndrome is a rare, neurodegenerative, autosomal recessive disease causing severe disability. Ataxia refers to poor coordination and telangiectasia to small, dilated blood vessels, both of which are hallmarks of the disease. Some have an increased number of respiratory tract infections (ear infections, sinusitis, bronchitis, and pneumonia) and according to the new classification of innate immunity errors (IEI), patients with A-T are included in group two of genetic syndromes with immunological involvement [9].

Besides that, A-T is a rare disease (OMIM 208,900) in which incidence ranges from 1: 40,000 to 1: 100,000, and it is caused by a mutation in the *ATM* gene (ataxia-telangiectasia mutated). The change induces difficulty in repairing ruptures of double-stranded DNA, increases ROS production, mitochondrial dysfunction, with concomitant oxidative stress and consequent cell apoptosis [10, 11]. DNA damage, combined with oxidative stress, can contribute to the pathogenesis of associated chronic diseases, including atherosclerosis [12, 13].

Due to advances in the care of patients, different studies reveal increasing survival rates, with ensuing concern about the possible appearance of further associated diseases such as cardiovascular [14, 15]. Dyslipidemia, a component of the metabolic syndrome, is one of the greatest risk factors with an impact on atherogenesis, which increases the risk of coronary failure two to three times [8]. To date, few studies evaluated the risk of atherosclerosis in patients with A-T [16, 17]. Especially in A-T patients there are no studies on the association of selenium and lipid status biomarkers.

The present study aims to describe the plasma levels of selenium and erythrocyte glutathione peroxidase activity in A-T patients and to relate them to oxidative stress and lipid status biomarkers.

## Methods

This is a cross-sectional controlled study evaluating 22 A-T patients of both genders, between 3 and 27 years of age, who were diagnosed with probable A-T (clinical phenotype classical) according to the criteria of the European Society for Immunodeficiencies (ESID) [18, 19].

The control group was recruited from a Primary Health Care Service and composed of 18 healthy volunteers matched by age and gender.

The Research Ethics Committee from the Federal University of São Paulo and financed by The São Paulo Research Foundation—FAPESP no 2015/13308-9.

The demographic, clinical, and treatment data were obtained from the patients’ charts. The family history risk of atherosclerosis was assessed for patients and controls.

At the time of sample collection, none of the subjects had an acute infectious disease, nor had they been using corticosteroids for at least 3 months; one patient was using antifungal drug and five were using antibiotics.

### Anthropometric assessment and food intake

The anthropometric assessment involved measurements of weight, height, mid-upper arm circumference (MUAC), and skinfold thickness (tricipital, subscapular, bicipital, and sacroiliac) as proposed by the World Health Organization (WHO) and Frisancho (1990) [20, 21]. The patients who were unable to stand upright had their weight measured in the wheelchair, on a specific scale for wheelchair-users (Micheletti-capacity 1100 lbs—serial: 2,161,058). Recumbent height was measured with the patient lying on a flat and firm surface, using an inextensible tape graduated in centimeters.

To assess the body mass index (BMI) and height for age (H/A) of children and adolescents, expressed as Z-scores, the WHO [22] criteria and the classification proposed by De Onis et al. were adopted [23]. For adults, the cut-off point of the WHO for BMI was used [20]. The sum of skinfold thickness and MUAC was used to estimate children’s body composition [24–26]. While for adults, the estimation of body composition was based on the sum of the four different skinfolds [27]. The body fat percentage was classified according to Deurenberg et al. and Lohman [25, 26].

The pubertal stage was evaluated according to Marshall and Tanner [28].

The assessment of food intake was performed using a 24 h dietary recall (R24hs), applied 3 times, with an interval of 15 days between them. The calculation of nutrients was performed using the Software Dietwin®, comparing the cases to the controls [29, 30].

Considering that food composition tables available in some software do not have complete data on Se content in food, these data were included manually based on the article by Ferreira et al. [31]. Only one of A-T patients had feeding tubes.

### Biochemical assessment

After 8-h fasting, blood was collected by peripheral venipuncture to analyze plasma selenium, erythrocyte glutathione peroxidase activity, lipid profile, apolipoproteins A-1 and B (Apo A-1, Apo B), oxidized LDL (LDLox), malondialdehyde (MDA), ultra-sensitive C-reactive protein (us-CRP), adiponectin, insulin, glucose, aspartate aminotransferase (AST), alanine transaminase (ALT), and gamma-glutamyl transpeptidase (Gamma GT).

All analyzes were performed using standard methods and good practice in clinical analysis. For determination of plasma selenium levels, the method used was atomic absorption spectrophotometry by graphite oven, with a detection limit of 1.0 mcg/L and linearity of 400.0 µg/L. The coefficient of variance was 0.8%. us-CRP was determined using the turbidimetric-immunological method (Roche). The measurement range is 1.0–200 mg/L. The variation coefficient was 1.3%. GPx activity was determined by the method based on that of Paglia and Valentine. (RANDOX). The method is linear up to a concentration of 925 U/L. The sensitivity was 75 U/L and the coefficient of variance was 3.4%. The lipid peroxidation was determined by the TBARS method (thiobarbituric acid-reactive substances) which is based on the reaction of malondialdehyde (MDA), a compound formed by the oxidation of lipids, with thiobarbituric acid (TBA) and is given in MDA equivalents, according to Satoh [32].

For classification of selenium levels, the cut-off point ≤ 46 µg/L was adopted for inadequacy. Glutathione peroxidase activity values lower than 4171 U/L were considered inadequate.

The lipid profile, including triglyceride, total cholesterol and high-density lipoprotein cholesterol (HDL-c) was measured by enzymatic-colorimetric tests. Low-density lipoprotein cholesterol (LDL-c) and very-low-density lipoprotein cholesterol (VLDL-c) were calculated using the formula by Friedewald et al. [33].

For classification, the cut-off points suggested by the American Academy of Pediatrics [34] and the National Cholesterol Education Program (NCEP) [35] were adopted. The presence of dyslipidemia was considered when the TC > 170 mg/dL for children/adolescents and > 200 mg/dL for adults and/or LDL-c > 110 mg/dL for children/adolescents and > 129 mg/dL for adults and/or triglycerides > 100 mg/dL for children/adolescents and > 150 mg/dL for adults and/or HDL-c < 35 mg/dL for children/adolescents, < 40 mg/dL for women and < 50 mg/dL for men.

The non-HDL-c (NHDL-c) values were obtained by subtracting the HDL-c rates from the TC levels and classified according to Bogalusa [36] and NCEP. The following atherogenic indices were also calculated: total cholesterol/ HDL-c, Apo B/Apo A-1, LDL-c/Apo B, LDL-c/HDL-c [37], HDL-c/Apo A-1 [38].

Apo A-1 and Apo B were measured using kits of turbidimetric methods for human Apo A-1 and Apo B (Roche, Indianapolis, IN, USA) and oxidized LDL, (ELISA)^PRO^ (Wuhan Fine Biological Technology Co, Wuhan, China).

Glycemia was measured by enzymatic reference method with hexokinase, while insulin was quantified by electrochemiluminescence. Using the fasting glucose and insulin values, the HOMA-IR (Homeostasis Model Assessment of Insulin Resistance) rate was calculated utilizing the following formula: HOMA-IR = fasting glucose (mmol/L) × fasting insulin (µU/mL)/22.5. HOMA-IR was considerably elevated when > 3.16 [39].

To evaluate cardiovascular risk we consider inflammatory, oxidative stress and lipid status biomarkers.

### Statistical analysis

The SPSS 25.0 statistical package was used for statistical analysis. In the descriptive and bivariate analysis, categorical variables were presented in absolute and percentage numbers, and compared using the Chi-square test or Fisher’s exact test. Most continuous variables showed a non-parametric distribution and was decided to present them as medians and interquartile intervals and compare them using the Mann–Whitney test. The correlation between glutathione peroxidase activity and selenium levels was evaluated with Spearman test (rho).

The area under the ROC curve (AUC) along with the corresponding 95% confidence interval (CI). One ROC curve was reported to assess the discriminatory power between the variables studied which had a difference between groups in the univariate analysis. Lastly, a multi-ROC curve was generated for evaluated the discriminatory power of variable related to oxidative stress (glutathione peroxidase activity, malondialdehyde levels and group) and lipid biomarkers (oxidized LDL, Non-HDL cholesterol, Apo A-1/HDL-c, Apo B/Apo A-1 and group) in relation to selenium levels (≤ 46 µg/L e > 46 µg/L).

For multivariate analysis was used linear regression (ENTER method) with selenium (logarithm) as the dependent variable. The independent variables included in the model were those that had clinical relevance and those that showed a statistically significant difference in the bivariate analysis, excluding those in which collinearity was detected (correlation > 0.8). Thus, the model was built with group, age, us C-reactive protein, oxidized LDL, malondialdehyde, apoliprotein B/apoliprotein A-1, glutathione peroxidase activity and alanine aminotransferase.

A *p*-value less than 0.05 was considered statistically significant.

## Results

The general characteristics of patients with A-T are shown in Table 1. Among the evaluated patients, male gender predominated 16 (72.7%). The median of current age, the onset of symptoms and the time since diagnosis were 12.2 years (8.5–20.9), 12.0 months (6.0–12.0) and 7.1 (4.4–13.1) years, respectively. At the time of evaluation, 12 (54.5%) and 5 (22.7%) patients were using intravenous immunoglobulin and prophylactic antibiotic therapy. Three A-T patients (13.6%) had pneumopathy.

**Table 1:** Characterization of A-T patients

Variable		N = 22
Age	Years	12.2 (8.5–20.9)^a^
Gender	Male	16 (72.7%)^b^
Onset of symptoms	Months	12.0 (6.0–12.0)^a^
Disease duration (since diagnosis)	Years	7.1 (4.4–13.1)^a^
Regular intravenous immunoglobulin	Yes	12 (54.5%)^b^
Pulmonar associated disease	Yes	3 (13.6%)^b^
Prophylactic antibiotic	Yes	5 (22.7%)^b^
Oral multivitamins	Yes	17 (77.3%)^b^
Family history of early cardiovascular disease	Yes	14 (63.6%)^b^
Alanine aminotransferase	> 40 U/L	5 (22.7%)^b^
AST/ALT	> 1	17 (77.3%)^b^

AST/ALT aspartate aminotransferase/alanine aminotransferase

^a^Median (IQ_25-75_). IQ—interquartile interval

^b^N (%)

In Table 2 shows there was no difference between the groups regarding age, gender, pubertal stage and body mass index. There was also no difference between groups regarding socioeconomic status and per capita income (data not shown). In turn, the A-T group had lower abdominal waist and lean body mass compared to the control group.

**Table 2:** Comparison of the variables: demographic, anthropometric, lipid status biomarkers, selenium, glutathione peroxidase activity, malondialdehyde, C-reactive protein of A-T patients and controls

	Variables	A-T patients (n = 22)	Controls (n = 18)	*P*-value
Age	Years	12.2 (8.5–20.9)	15.8 (9.8–22.8)	0.615^a^
Gender	Male	16 (72.7%)	13 (72.2%)	0.972^b^
Prepubertal	Yes	6 (27.3%)	3 (16.7%)	0.506^b^
Body mass index (kg/m^2^)	Underweight	9 (40.9%)	2 (11.1%)	0.146^b^
	Normal	11 (50.0%)	14 (77.8%)	
	Overweight	2 (9.1%)	2 (11.1%)	
Waist circumference	cm	60.0 (53.0–64.0)	68.8 (61.0–79.0)	0.004^a^
Lean body mass	kg	24.8 (20.2–29.8)	41.8 (31.0–50.4)	0.001^a^
Dyslipidemia	Yes	14 (63.6%)	11 (78.6%)	0.467^b^
Lipid profile	High CT	13 (59.1%)	4 (28.6%)	0.097^b^
	High LDL-c	12 (54.4%)	1 (7.1%)	0.005^b^
	Low HDL-c	4 (18.2%)	6 (42.8%)	0.140^b^
	High triglycerides	5 (22.7%)	1 (7.1%)	0.370^b^
	High non-HDL-c	13 (59.1%)	2 (14.3%)	0.014^b^
Glycemia	Elevate (> 100 mg/dL)	1 (4.5%)	1 (7.1%)	0.633^b^
HOMA-IR	Elevate (> 3.16)	6 (27.3%)	2 (14.3%)	0.441^b^
Selenium	Adequate (> 46 μg/L)	13 (59.1%)	11 (78.6%)	0.292^b^
Glutathione peroxidase	Adequate (> 4171 U/L)	20 (90.9%)	11 (78.6%)	0.357^b^
Malondialdehyde	High (≥ 3.46 nmol/mL)	16 (72.7%)	5 (35.7%)	0.041^b^
C-reactive protein	High (> 3 mg/L)	8 (36.4%)	1 (7.1%)	0.062^b^

Laboratory variables in control group (N = 14)

Significance level of: ^a^Mann-Whitney test, Median (IQ_25-75_). ^b^Chi-square test, N (%)

CT, total cholesterol; LDL-c, low density lipoprotein cholesterol; HDL-c, high density lipoprotein cholesterol; NHDL-c, Non-HDL-c; HOMA-IR, homeostasis model assessment of insulin resistance

Adequate levels of selenium were observed in 24/36 (66.7%) in this evaluated population. There was no statistically significant difference between the groups in selenium levels, either in stratified (Adequate, > 46 μg/L: 59.1% vs 78.6%, *p* = 0.292) or continuous form [47.6 μg/L (43.2–57.0) vs 54.6 (45.2–62.6) μg/L, *p* = 0.242] (Table 2 and Table 3). The A-T group showed a higher percentage of inadequacy for LDL-c (54.4% vs 7.1%, *p* = 0.005), non-HDL-c (59.1% vs 14.3%, *p* = 0.014) and malondialdehyde serum levels (72.7% vs 35.7%, *p* = 0.041) (Table 2). More than 60% of A-T patients had dyslipidemia. There was no correlation between AFP concentrations and lipid status biomarkers (data not shown).

**Table 3:** Comparison of biochemical variables of A-T patients and controls

Variables		Units		A-T patients (n = 22)	Controls (n = 18)	*P*-value
*Lipid status biomarkers*						
Total cholesterol		mg/dL		188.5 (168.0–228.0)	164.0 (124.0–172.0)	0.005
LDL-c		mg/dL		124.4 (105.4–166.0)	89.5 (67.0–105.6)	< 0.001
Triglycerides		mg/dL		90.5 (66.0–115.0)	77.5 (63.0–94.0)	0.231
HDL-c		mg/dL		47.5 (42.0–61.4)	46.0 (35.0–56.0)	0.549
Non-HDL-c		mg/dL		136.5 (120.0–182.0)	108.0 (79.0–125.0)	0.001
VLDL-c		mg/dL		18.1 (13.2–23.0)	15.5 (12.6–18.8)	0.231
Remnant cholesterol		mg/dL		18.2 (13.2–23.0)	15.6 (12.6–18.8)	0.221
Oxidized LDL		mg/dL		72.6 (42.6–137.0)	25.8 (21.1–40.7)	< 0.001
Apolipoprotein A-1		mg/dL		99.0 (87.0–117.0)	122.0 (92.0–138.0)	0.070
Apoliprotein B		mg/dL		109.5 (96.0–138.0)	91.0 (78.0–101.0)	0.002
Apoliprotein B/Apoliprotein A-1				1.1 (0.7–1.5)	0.7 (0.5–0.8)	0.001
Total cholesterol/HDL-c				4.0 (3.0–5.0)	3.0 (3.0–4.0)	0.080
LDL-c/Apoliprotein B				1.1 (1.0–1.2)	1.1 (0.9–1.1)	0.549
LDL-c/ HDL-c				2.5 (2.0–4.0)	2.0 (1.0–2.0)	0.021
Triglycerides/HDL-c				2.0 (1.0–3.0)	1.0 (1.0–3.0)	0.279
Apoliprotein A-1/HDL-c				1.9 (1.8–2.1)	2.5 (2.2–2.7)	0.008
*Oxidative stress and inflammatory biomarkers*						
Glutathione peroxidase		U/L		7300 (6683–8267)	8686 (7967–9449)	0.005
Selenium		µg/L		47.6 (43.2–57.0)	54.6 (45.2–62.6)	0.242
Malondialdehyde		nmol/mL		3.8 (3.0–4.0)	2.8 (2.4–3.6)	0.029
C-reactive protein		mg/L		1.0 (0.3–9.8)	0.65 (0.3–1.6)	0.116
Adiponectin		mg/dL		156.9 (125.5–234.6)	180.4 (173.1–183.9)	0.355
*Glucose metabolism biomarkers*						
Blood glucose		mg/dL		85 (77.0–88.5)	83.0 (77.2–90.0)	0.810
Insulin		uU/mL		6.1 (2.5–11.6)	7.4 (3.6–10.6)	0.870
HOMA-IR				1.1 (0.4–3.4)	1.2 (0.7–2.0)	0.891
*Liver enzymes*						
Aspartate aminotransferase		U/L		31.0 (25.0–47.0)	17.0 (14.0–19.0)	< 0.001
Alanine aminotransferase		U/L		21.0 (13.0–32.0)	9.5 (9.0–13.0)	< 0.001
Gamma-glutamyl transpeptidase		U/L		27.7 (14.3–66.7)	14.6 (10.1–17.4)	0.011

Laboratory variables in control group (N = 14)

Significance level of Mann–Whitney test, Median (IQ_25-75_)

LDL-c, low density lipoprotein cholesterol; HDL-c, high density lipoprotein cholesterol; VLDL-c, very low density lipoprotein cholesterol; HOMA-IR, homeostasis model assessment of insulin resistance

Table 3 compared the markers of the lipid profile, oxidative stress, inflammatory and serum levels of liver enzymes between the groups. The A-T group presented, in comparison to the control group, higher levels of LDL-c, non-HDL-c, oxidized LDL, Apo B, Apo B/Apo A-1, LDL-c/HDL-c ratio and lower Apo A-1/HDL-c. It was also observed that the A-T group had lower levels of glutathione peroxidase activity [7300 U/L (6683–8267) vs 8686 U/L (7967–9449), *p* = 0.005], higher malondialdehyde [3.8 µg/L (3.0–4.0) vs 2.8 µg/L (2.4–3.6), *p* = 0.029] and liver enzymes.

There was no statistically significant correlation between selenium levels and glutathione peroxidase activity in the whole group (rho = 0.161, *p* = 0.347), A-T group (rho = 0.048, *p* = 0.832) and control group (rho =  − 0.011, *p* = 0.970).

**Fig. 1:**
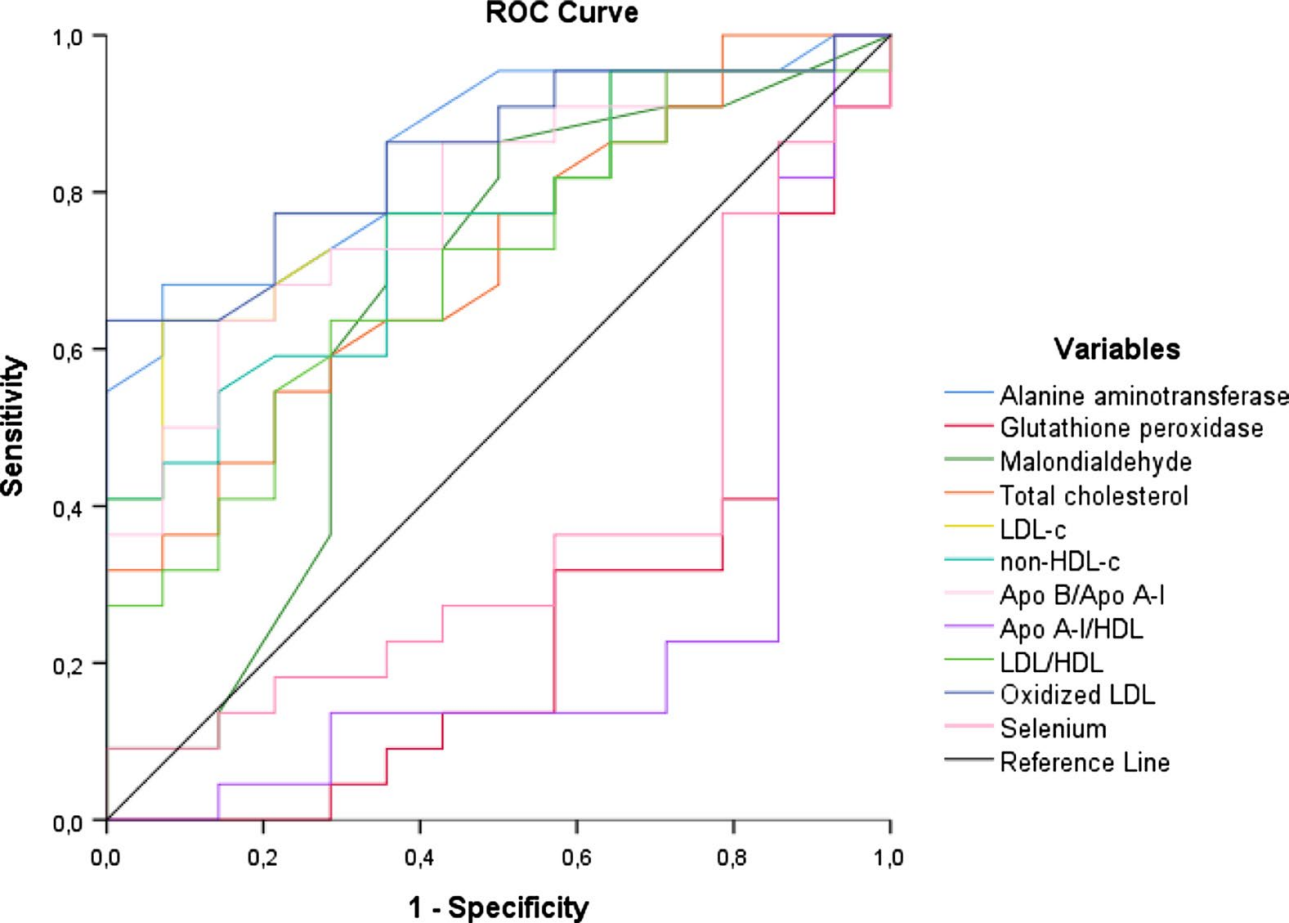
Discriminatory analysis using the ROC curve of the biochemical variables evaluated in A-T patients and the control group

As shown in the ROC curve (Fig. 1), oxidized LDL (AUC = 0.849, 95% CI 0.725–0.973) and ALT (AUC = 0.854, 95% CI 0.732–0.976) were the variables that showed the greatest discriminatory power between groups.

Through linear regression that included in the model: group (A-T), age (years), ALT (U/L), C-reactive protein (mg/L), non-HDL-c (mg/dL), Apo B ratio/Apo A-1, malondialdehyde (nmol/L), glutathione peroxidase activity (U/L) and oxidized LDL (mg/dL), it was found that selenium levels were influenced, in both groups, independently only by the concentrations of oxidized LDL, malonaldehyde and non-HDL-c (Table 4).

**Table 4:** Biochemical variables associated to selenium levels in A-T patients and controls (n = 36)

Variables		B	*P*-value	Confidence interval 95%	
Malondialdehyde (log)	nmol/L	− 0.583	0.001	− 0.909	− 0.258
LDL-oxidized (log)	mg/dL	− 0.180	0.021	− 0.331	− 0.030
Glutathione peroxidase (log)	U/L	− 0.166	0.175	− 0.410	0.078
Apo B/Apo A-1		− 0.057	0.154	− 0.138	0.023
Group	AT	− 0.035	0.499	− 0.138	0.069
Age	Years	− 0.002	0.420	− 0.008	0.003
Non-HDL-c	mg/dL	0.002	0.003	0.001	0.003
C-reactive protein (log)	mg/L	0.006	0.833	− 0.049	0.060
Alanine aminotransferase (log)	U/L	0.100	0.161	− 0.043	0.243

R^2^ = 0.548

Dependent variable: selenium μg/L (log—logarithmic)

**Fig. 2:**
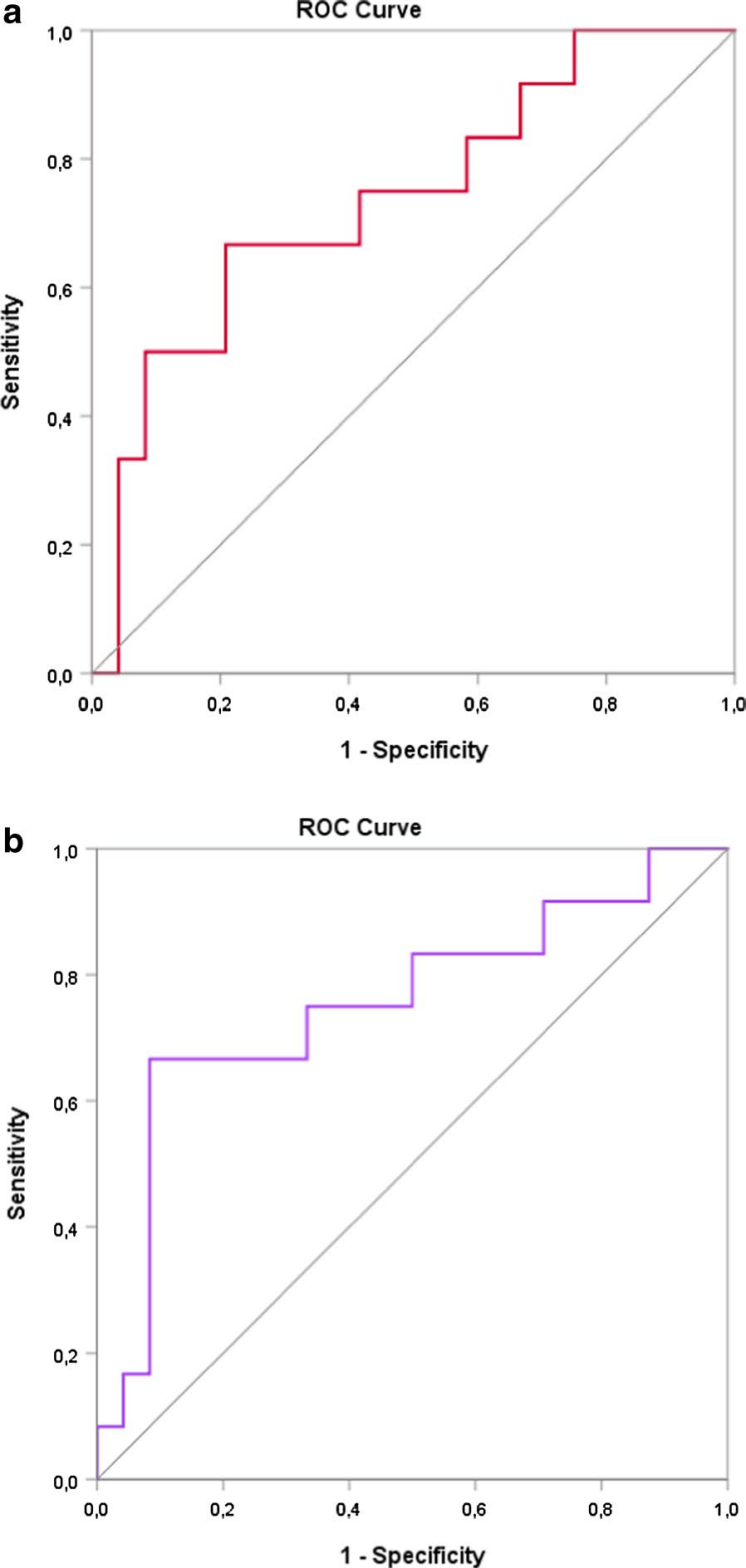
Discriminatory analysis using a multivariate ROC analysis of variables related to oxidative stress (**a**) and lipid status (**b**) with selenium levels (≤ 46 µg/L or > 46 µg/L)

The area under the multi-ROC curve for variables related to oxidative stress and lipid biomarkers in relation to selenium levels was 0.736 (95% CI 0.559–0.913) and 0.753 (95% CI 0.570–0.937), which is an acceptable level of discrimination (Fig. 2a, b).

A lower intake of energy, protein, carbohydrate was observed in A-T group, compared to control group. The intake of polyunsaturated, monounsaturated, trans fat and selenium was lower in A-T group (Table 5).

**Table 5:** Comparison of the means of energy, macronutrients, and micronutrients intake in A-T patients and controls

	A-T patients N = 22	Controls N = 18	*P*-value
Energy (Kcal)	1522.4 (1233.8–1673.1)	1744.1 (1431.0–2333.2)	0.031
Carbohydrate (g)	211.1 (187.8–249.6)	255.4 (218.4–354.3)	0.029
Protein (g)	60.3 (51.2–68.2)	76.8 (60.3–93.2)	0.010
Total fat (g)	42.5 (37.2–47.3)	44.1 (38.4–60.1)	0.127
Saturated fat (g)	13.9 (10.8–16.7)	17.1 (12.4–23.7)	0.068
Monounsaturated fat (g)	10.3 (6.3–11.6)	12.2 (11.2–15.4)	0.007
Polyunsaturated fat (g)	5.6 (4.0–7.6)	9.8 (6.6–13.5)	0.001
Trans fat (g)	0.5 (0.3–0.7)	0.9 (0.7–1.9)	0.006
Copper (mg)	0.6 (0.5–0.8)	0.6 (0.5–0.8)	0.913
Selenium (µg)	56.1 (43.2–66.0)	68.8 (63.0–80.1)	0.016
Zinc (mg)	7.1 (5.9–8.9)	6.0 (4.5–7.1)	0.074
Retinol (µg)	249.5 (176.8–395.0)	208.9 (150.5–247.2)	0.772
Ascorbic acid (mg)	74.6 (57.3–100.7)	57.6 (52.4–77.2)	0.142

Significance level of Mann–Whitney test, Median (IQ_25-75_)

## Discussion

The present study showed that selenium concentrations did not differ significantly between patients and controls. However, it must be pointed out that 41% of A-T patients had selenium concentrations below the reference value. MDA levels were higher and GPx activity lower in A-T patients compared to controls. Also A-T patients had an atherogenic lipid status.

To our knowledge, there are no current publications evaluating the association between selenium levels and GPx activity with lipid status biomarkers in A-T patients. In vitro study show that A-T cells are under a continuous oxidative stress and have an abnormal response to agents inducing this state [40]. Oxidative stress is a central mechanism in the pathogenesis of the disease, especially neurodegeneration [41] and other morbidities, such as liver disorders and dyslipidemias [16, 42].

An experimental study by Mercer et al. [43] demonstrated that ATM protein deficiency accelerates the atherosclerotic process via systemic effects (regulation of NF-κB expression) and on the vascular endothelium. The authors concluded that damage to mitochondrial DNA, increased production of free radicals and reduced oxidative phosphorylation, effects which are directly related to changes in lipid and glucose metabolism and present in A-T patients.

Squadrone et al. [44] evaluated the concentrations of trace elements, including selenium and the antioxidant enzymes, in 16 A-T patients and controls. They also found no differences in selenium levels, similarly to what we found in this study. In our study, GPx activity were lower in A-T patients; a finding not observed by Squadrone et al. The synthesis and activity of glutathione can be reduced when there is malnutrition, hyperglycemia, corticotherapy, skeletal muscle inactivity; situations frequently observed in A-T patients [45]. Some hypotheses may explain the higher MDA values and the lower GPx activity, pointing to an oxidative stress in our patients: (a) older age (median 12.2 years) (b) a significant change in liver enzymes, malnutrition, and impaired lean mass variables not mentioned in the study by Squadrone et al.

In this study A-T patients demonstrated an atherogenic profile, with emphasis on elevating NHDL-c and LDLox. LDL-c is easily susceptible to oxidation under conditions of oxidative stress, as confirmed in our study by the elevation of MDA (lipid peroxidation marker) and reduction of GPx activity in A-T patients; LDLox has some atherogenic characteristics. A recent metanalysis and systematic review described, based on observational studies, the association between LDLox concentrations and the development of atherosclerotic disease [46].

The findings of lower selenium intake in the A-T group combined with about 40% of serum levels below the reference value may complicate the neurodegeneration. Se is vital for the central nervous system and it is involved in various functions, such as motor performance, coordination, memory and cognition. The role of Se and selenoproteins in neurotransmission goes beyond their antioxidant properties and involves the regulation of inflammation. Furthermore, it plays a direct role in signaling by means of selenoprotein P and its interaction with 2 synaptic post receptors of Apolipoprotein (ApoER2) [47]. Future studies evaluating the role of selenium in neurodegeneration-related outcomes in A-T patients appear promising.

Regarding the association of selenium with total cholesterol and LDL-c, some studies show a direct association in the general population [48, 49], while others display a negative correlation [50, 51] or no association at all [52]. This inconsistency may be due to the sample size or even the lack of adjustment for confounding variables. In our study, we observed, after linear regression, that selenium levels were independent and inversely associated, in both groups, with concentrations of oxidized LDL, malonaldehyde and directly with non-HDL-c (Table 4).

An acceptable level of discrimination for variables related to oxidative stress and lipid status in relation to selenium levels emphasizes the importance of future studies assessing the impact of selenium supplementation on A-T patients.

In conclusion, we observed the presence of selenium below the reference value in nearly 40% and low GPx activity in A-T patients.

There was a significant, inverse and independent association between selenium concentrations and oxidative stress biomarkers. Those data reinforce the importance of assessing the nutritional status of selenium in those patients.

## Data Availability

The datasets used and/or analysed during the current study are available from the corresponding author on reasonable request.

## Abbreviations

AFP: Alpha-fetoprotein
ALT: Alanine transaminase
Apo B: Apolipoproteins B
Apo-A1: Apolipoproteins A-1
AST: Aspartate aminotransferase
A-T: Ataxia-telangiectasia
ATM: Ataxia-telangiectasia mutated
BMI: Body mass index
CVDs: Cardiovascular diseases
FAPESP: São Paulo Research Foundation
GGT: Gamma-glutamyl transpeptidase
GPx: Glutathione peroxidase activity
H/A: Height for age
HD: Heart disease
HDL-c: High-density lipoprotein cholesterol
HOMA-IR: Homeostasis Model Assessment of Insulin Resistance
IEI: Inborn errors of immunity
IQ: Interqartile interval
IVIG: Intravenous immunoglobulin
LDL-c: Low-density lipoprotein cholesterol
LDLox: Oxidized LDL
MDA: Malondialdehyde
MUAC: Mid-upper arm circumference
NCEP: National Cholesterol Education Program
NFkB: Nuclear transcription factor
NHDL-c: Non-HDL-c
ROS: Reactive oxygen species
Se: Selenium
TC: Total cholesterol
TG: Triglycerides
us-CRP: Ultra-sensitive C-reactive protein
VLDL-c: Very-low-density lipoprotein cholesterol
